# Supplementary material for: Melatonin attenuates kidney injury by alleviating lysosomal damage in diabetic kidney disease: Melatonin delays DKD by improving lysosomal injury
Source: Acta Biochim Biophys Sin (Shanghai). 2025 Jun 13;57(10):1589–600. doi: 10.3724/abbs.2025034 (PMC12616729; doi:10.3724/abbs.2025034)
Supplement: 24607Supplementary_Data [file 24607Supplementary_Data.docx]

**Supplementary Table S1. The sequences of TFEB siRNA**

| Identification of product | TFEB siRNA | Sequence (5′→3′) |
| --- | --- | --- |
| stB0008444A | genOFFTM st-h-TFEB_001 | GACGAAGGTTCAACATCAA |
| stB0008444B | genOFFTM st-h-TFEB_002 | GAAAGACAATCACAACTTA |
| stB0008444C | genOFFTM st-h-TFEB_003 | GCAACAGGCTGTCATGCAT |

**Supplementary Table S2. Sequences of primers used for quantitative RT-PCR**

| Host | Gene | Primer sequence (5′→3′) |
| --- | --- | --- |
| Human | *LRP-1* | F: GGCTGATCAGGTGTCGGAAA  R: GTCACCGTTGTTGACACTGC |
|  | *MMD* | F: ATGGAATGGGACTCTGTGCC |
|  |  | R: CAGCTGCCATGAGCCAGATA |
|  | *NSF* | F: TGCTGGTGCAGCAGACTAAG |
|  |  | R: TCATGGCCTGACATTTGGCT |
|  | *TFEB* | F: TCTGTCCAGCAACATGACAGC |
|  |  | R: GGATGTGGGATTCTCCAGGT |
| Mouse | *LRP-1* | F: GCGGTGTGACAACGACAATG |
|  |  | R: GGTCTTGTAGCCTGGTTGGT |
|  |  | R: TCTATCGGATACTTCAGCGTCA |

**Supplementary Table S3. The sequence of wild-type or mutant LRP-1 3**′**UTR**

| Plasmid | h- LRP-1 ENST00000243077.3 3′UTR-WT |
| --- | --- |
| Sequence | ctcgagGgccctgccccgtcggactgcccccagaaagcctcctgccccctgccagtgaagtccttcagtgagcccctccccagccagcccttccctggccccgccggatgtataaatgtaaaaatgaaggaattacattttatatgtgagcgagcaagccggcaagcgagcacagtattatttctccatcccctccctgcctgctccttggcacccccatgctgccttcagggagacaggcagggagggcttggggctgcacctcctaccctcccaccagaacgcaccccactgggagagctggtggtgcagccttcccctccctgtataagacactttgccaaggctctcccctctcgccccatccctgcttgcccgctcccacagcttcctgagggctgcggccgc |
| Plasmid | h- LRP-1 ENST00000243077.3 3′UTR-MUT |
| Sequence | ctcgagGgccctgccccgtcggactgcccccagaaagcctcctgccccctgccagtgaagtccttcagtgagcccctccccagccagcccttccctggccccgccggatgtataaatgtaaaaaggcatggattacattttatatgtgagcgagcaagccggcaagcgagcacagtattatttctccatcccctccctgcctgctccttggcacccccatgctgccttcagggagacaggcagggagggcttggggctgcacctcctaccctcccaccagaacgcaccccactgggagagctggtggtgcagccttcccctccctgtataagacactttgccaaggctctcccctctcgccccatccctgcttgcccgctcccacagcttcctgagggctgcggccgc |

The sequence labeled in yellow is the binding site of LRP-1 3'UTR to miR-205-5p.

**Supplementary Table S4. The sequence of wild-type or mutant miR-205-5p promoter**

| Plasmid | h-miR-205-5p promoter hg38_dna range=chr1:209426820-209428820 promoter-WT |
| --- | --- |
| Sequence | gctagcGTGAATGAAATCCACAGGCCCATCACAAGATGGCTGCGCTCTCCAGTCCCCGGCACCCCAGGCTGCTCTGAACATGTGAGAGCTCCTCCCTCAAGGCCTGAATTTTCGCAGGTCTTCATCACACTCAGCCCGCCATCATTTTTTCAGATTCAGTTCCAATATGTCACTCTGTCTGTAGAGCCTTCCCTGAATCTCACAGACAAATGCGGCAGTTCCCTTTGTGGGGGATATTCATCATGTTTTTCCTCATTGGCCTATGTTTTTGCCTCTTCTTGTAGATGTCCATCTAGGCTGGGAGCTCCTAGGGGGTAAACGCAGCCTCTCACTCACCTGGCACAGAGTCGATGCTCATGGAATGATACGTGATGGGGAGAGTGAGTAAGTGAATGAATGAATGAATGCTATTCTGGAAGGAAGAGGGAGAGAGGGAGCAGGGAAAGGTCAGAAGGGGGCCCAGAGTCTTCAGCACACAATGTGGGTGTATCACTGTGGCCAGGAACAATTCGCTTCTCTGCCTGCATTACTTTTTGGGAAATGGGAGAGCTGAGGCTCAGTGTAGGCAAACTGGGTCCAGTCAGCAGGGAAGCCATGAGCTCCACGGTTTAGGGGAGGAGTCCGGTGCTTAAGGAAGCTATCACGTGGAAGATCTCCCTGCCCTCCTCCTATTGGTCTGTATTGGTTTTTCTTCTTCACCAGTGGAGGCTTTTCTTCTCCCAGGAAGAATCCAGAATAGTTCAGACAAGCTTCAGGTCCGCCAGAACCGGGGAAAACAACGTGTAGGGTTTGTTTTAAAGGGCTTTTAAATGGGGTTTGGGAGATCCAGGTAGATTAGAATCTTGACTCTGCACTGGCTGTGCAAGACAGAAATTCACCTTTCAAAACTTCTGTTCCCCAGTCTATGCGGTAAGAAGTTTAGATTAGCTGATTTCTAGGGAGATTTTGGGCAGGCATGATTCAGTTCCATGAAGGGCTGTGGGACAGGACTGCACTAAGAGGTCCTATAGATTGGATTCTTCAGTTCCCTCCCTTCCGCTGGAGAATAGAGGAATCTGCCTTCGCTGCACACTAGATTTACCTGGAGAACAAATGGAAAGCAGGCCAGGATCCTGGGTGAAATGTGGCACATTTGAGGAGACTTCAACTTCCCTTGCCCTCCAGGTGTGCACTTGGAAGGGGGAACGAGGGAGGGGGAAGCTGGCAAGATGGGCCGAGAGATAGGGGAGGGGCAGGAGGCGGAGCCCAAGTTGCGCATGGAAGCGGGGGTGGGGTAAAATAATCAAGTTTATAGACCGCCCTCTTTAAAGTTACTAATGAGCTTGCCTTCTCTTTCCTTAATTTCCCCTCGCAGTGTGGTCTTTTCCCCACCCCCAGACATGAAAGGGAAGCAGGTCACAAAGCCTTTCGGATTATAAAAGAAACACTTGCTTCTCACAAGGGGAGCAGCAGACTTACTCTGTACTAAATGCCAGGATAAGCCTCTGGCTGGGCCTCGACTGTGACCCTCCGGCCTCTTTCTACAGCTCTGCCTGGATGGACTGGCCTATCTCTGCTGGATTCCCGAAGTGCATTGTGTAGAGACAGCAACTCAGGTCAGGCTAAAAGCTCAAGCAAGCAAGCGCGCACACACACGCGCGCACACACACACACACACACACAAACACTCAGCTTCCTTAGGACAAGATAAAATCTTAGCATTCCCCTCTCCCCGATTAGGTAGGTCTCTGGGAGGACTAAGGCTTCAGGTGCAAGGCTCAGATAACCTGCAGTGTCTCTCCAACTCTGGGATGACAAAGACCTCACTTCCCTTTTCTGGTGTTCACACAACAATGAGAAAGTACGGGTAGCCTGCAGAAAGACCTCTCCATTCATGGTCCCCCAGGGGTGTGGGTTCTGAGAGGTGGGACCAGCTGCCAGGCCCTTTCTCCATTGGTTGAGTTCAGCAGGTAACCTGAAGCTTTGCTGAGAGGTGCATAAATAAAGAGTGAAACTAGTACCACCTCCgtcgac |
| Plasmid | h-miR-205-5p promoter hg38_dna range=chr1:209426820-209428820 promoter-MUT |
| Sequence | gctagcGTGAATGAAATCCACAGGCCCATCACAAGATGGCTGCGCTCTCCAGTCCCCGGCACCCCAGGCTGCTCTGAACATGTGAGAGCTCCTCCCTCAAGGCCTGAATTTTCGCAGGTCTTCATCACACTCAGCCCGCCATCATTTTTTCAGATTCAGTTCCAATATGTCACTCTGTCTGTAGAGCCTTCCCTGAATCTCACAGACAAATGCGGCAGTTCCCTTTGTGGGGGATATTCATCATGTTTTTCCTCATTGGCCTATGTTTTTGCCTCTTCTTGTAGATGTCCATCTAGGCTGGGAGCTCCTAGGGGGTAAACGCAGCCTCTCACTCACCTGGCACAGAGTCGATGCTCATGGAATGATACGTGATGGGGAGAGTGAGTAAGTGAATGAATGAATGAATGCTATTCTGGAAGGAAGAGGGAGAGAGGGAGCAGGGAAAGGTCAGAAGGGGGCCCAGAGTCTTCAGCACACAATGTGGGTGTATCACTGTGGCCAGGAACAATTCGCTTCTCTGCCTGCATTACTTTTTGGGAAATGGGAGAGCTGAGGCTCAGTGTAGGCAAACTGGGTCCAGTCAGCAGGGAAGCCATGAGCTCCACGGTTTAGGGGAGGAGTCCGGTGCTTAAGGAAGCTTaCgCtTaAcAGATCTCCCTGCCCTCCTCCTATTGGTCTGTATTGGTTTTTCTTCTTCACCAGTGGAGGCTTTTCTTCTCCCAGGAAGAATCCAGAATAGTTCAGACAAGCTTCAGGTCCGCCAGAACCGGGGAAAACAACGTGTAGGGTTTGTTTTAAAGGGCTTTTAAATGGGGTTTGGGAGATCCAGGTAGATTAGAATCTTGACTCTGCACTGGCTGTGCAAGACAGAAATTCACCTTTCAAAACTTCTGTTCCCCAGTCTATGCGGTAAGAAGTTTAGATTAGCTGATTTCTAGGGAGATTTTGGGCAGGCATGATTCAGTTCCATGAAGGGCTGTGGGACAGGACTGCACTAAGAGGTCCTATAGATTGGATTCTTCAGTTCCCTCCCTTCCGCTGGAGAATAGAGGAATCTGCCTTCGCTGCACACTAGATTTACCTGGAGAACAAATGGAAAGCAGGCCAGGATCCTGGGTGAAATGTGGCACATTTGAGGAGACTTCAACTTCCCTTGCCCTCCAGGTGTGCACTTGGAAGGGGGAACGAGGGAGGGGGAAGCTGGCAAGATGGGCCGAGAGATAGGGGAGGGGCAGGAGGCGGAGCCCAAGTTGCGCATGGAAGCGGGGGTGGGGTAAAATAATCAAGTTTATAGACCGCCCTCTTTAAAGTTACTAATGAGCTTGCCTTCTCTTTCCTTAATTTCCCCTCGCAGTGTGGTCTTTTCCCCACCCCCAGACATGAAAGGGAAGCAGGTCACAAAGCCTTTCGGATTATAAAAGAAACACTTGCTTCTCACAAGGGGAGCAGCAGACTTACTCTGTACTAAATGCCAGGATAAGCCTCTGGCTGGGCCTCGACTGTGACCCTCCGGCCTCTTTCTACAGCTCTGCCTGGATGGACTGGCCTATCTCTGCTGGATTCCCGAAGTGCATTGTGTAGAGACAGCAACTCAGGTCAGGCTAAAAGCTCAAGCAAGCAAGCGCGCACACACACGCGCGCACACACACACACACACACACAAACACTCAGCTTCCTTAGGACAAGATAAAATCTTAGCATTCCCCTCTCCCCGATTAGGTAGGTCTCTGGGAGGACTAAGGCTTCAGGTGCAAGGCTCAGATAACCTGCAGTGTCTCTCCAACTCTGGGATGACAAAGACCTCACTTCCCTTTTCTGGTGTTCACACAACAATGAGAAAGTACGGGTAGCCTGCAGAAAGACCTCTCCATTCATGGTCCCCCAGGGGTGTGGGTTCTGAGAGGTGGGACCAGCTGCCAGGCCCTTTCTCCATTGGTTGAGTTCAGCAGGTAACCTGAAGCTTTGCTGAGAGGTGCATAAATAAAGAGTGAAACTAGTACCACCTCCgtcgac |

The sequence labeled in yellow is the binding site of miR-205-5p promoter to TFEB.


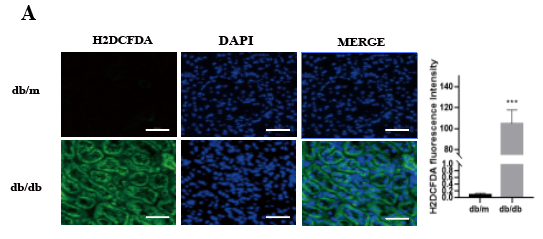


**Supplementary Figure S1** **Fluorescence intensity of ROS in db/db and db/m mice**  ****P* < 0.001 vs the db/m group (n = 5/group).


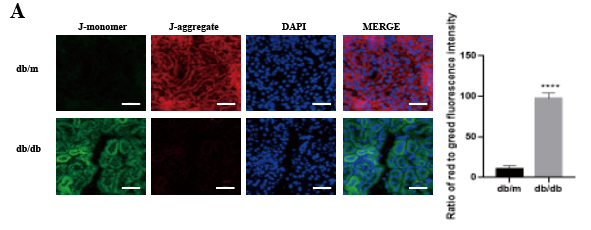


**Supplementary Figure S2.** **JC-1 staining images in db/db and db/m mice**  *****P* < 0.0001 vs the db/m group (*n* = 5/group).


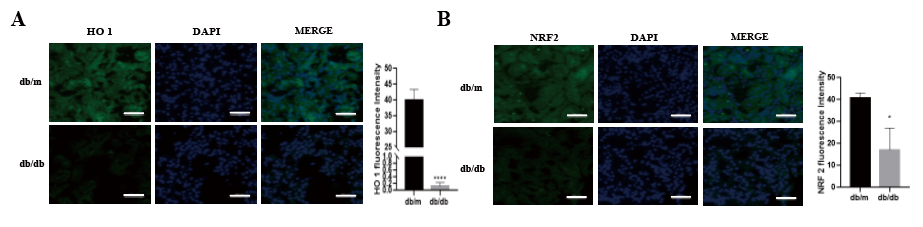


**Supplementary Figure S3.** **Immunofluorescence staining for HO 1 and NRF2 in db/db and db/m mice** (A) Immunofluorescence staining for HO 1 in db/db and db/m mice. *****P* < 0.0001 vs the db/m group (*n* = 5/group). (B) Immunofluorescence staining for NRF2 in db/db and db/m mice. **P* < 0.05 vs the db/m group (*n* = 5/group).


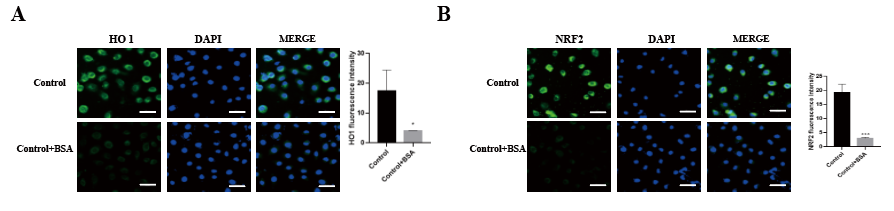


**Supplementary Figure S4.** **Immunofluorescence staining for HO 1 and NRF2 in BSA-HK-2 cells and control cells** (A) Immunofluorescence staining for HO 1 in BSA-HK-2 cells and control cells. **P* < 0.05 vs the control group (*n* = 3/group). (B) Immunofluorescence staining for NRF2 in BSA-HK-2 cells and control cells. ****P* < 0.001 vs the control group (*n* = 3/group).


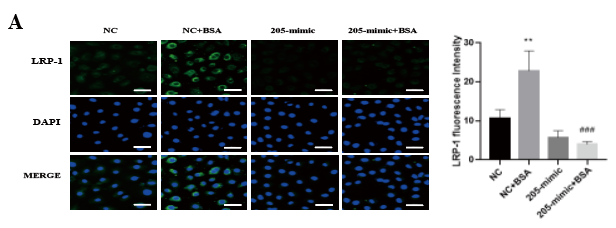


**Supplementary Figure S5.** **Immunofluorescence staining for LRP-1 in miR-205 mimic-treated HK-2 cells**  ***P* < 0.01 vs the NC group. ^###^*P* < 0.001 vs the miR-205 mimic group (*n* = 5/group).


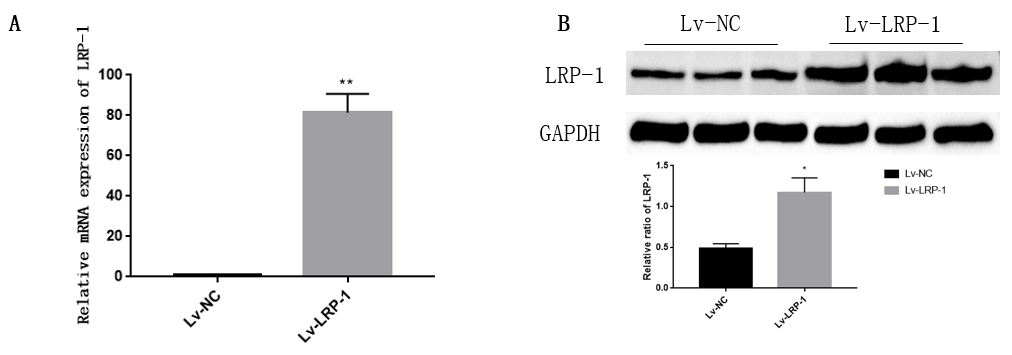


**Supplementary Figure S6.** **LRP-1 mRNA and protein expressions in HK-2 cells transfected with the pEZ-Lv201-LRP-1 plasmid** (A) Relative *LRP-1* mRNA expression in HK-2 cells transfected with the pEZ-Lv201-LRP-1 plasmid. ***P* < 0.01 compared with the Lv-NC group (*n* = 3/group). (B) Relative LRP-1 protein levels in HK-2 cells transfected with the pEZ-Lv201-LRP-1 plasmid. **P* < 0.05 compared with the Lv-NC group (*n* = 3/group).


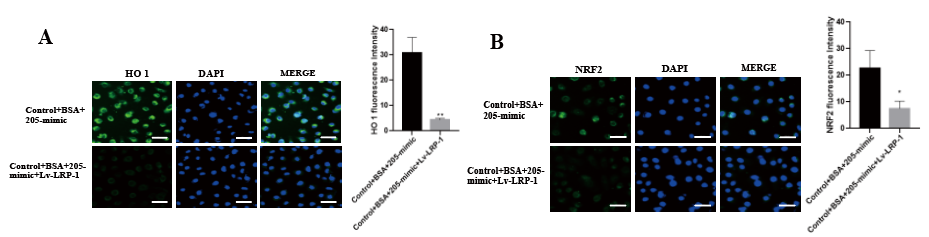


**Supplementary Figure S7.** **Immunofluorescence staining for HO 1 (A) and NRF2 (B) in BSA-HK-2 cells transfected with a miR-205-5p mimic with or without concomitant LRP-1 overexpression** (A) Immunofluorescence staining for HO 1 in BSA-HK-2 cells transfected with a miR-205-5p mimic with or without concomitant LRP-1 overexpression. ∗∗*P* < 0.01 vs. BSA-HK-2 cells transfected with a miR-205-5p mimic without concomitant LRP-1 overexpression (n=3/group). (B) Immunofluorescence staining for NRF2 in BSA-HK-2 cells transfected with a miR-205-5p mimic with or without concomitant LRP-1 overexpression. **P* < 0.05 vs BSA-HK-2 cells transfected with a miR-205-5p mimic without concomitant LRP-1 overexpression (*n* = 3/ group).


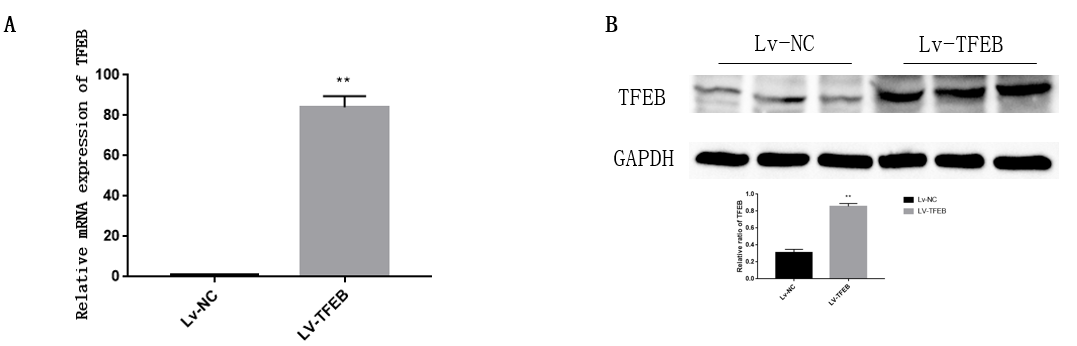


**Supplementary Figure S8.** **TFEB mRNA and protein expressions in HK-2 cells transfected with the pEZ-Lv201-TFEB plasmid** (A) Relative *TFEB* mRNA expression in HK-2 cells transfected with pEZ-Lv201-TFEB plasmid. ***P* < 0.01 compared with the si-NC group (*n* = 3/ group). (B) Relative TFEB protein levels in HK-2 cells transfected with pEZ-Lv201-TFEB plasmid. ***P* < 0.01 compared with the si-NC group (*n* = 3/group).


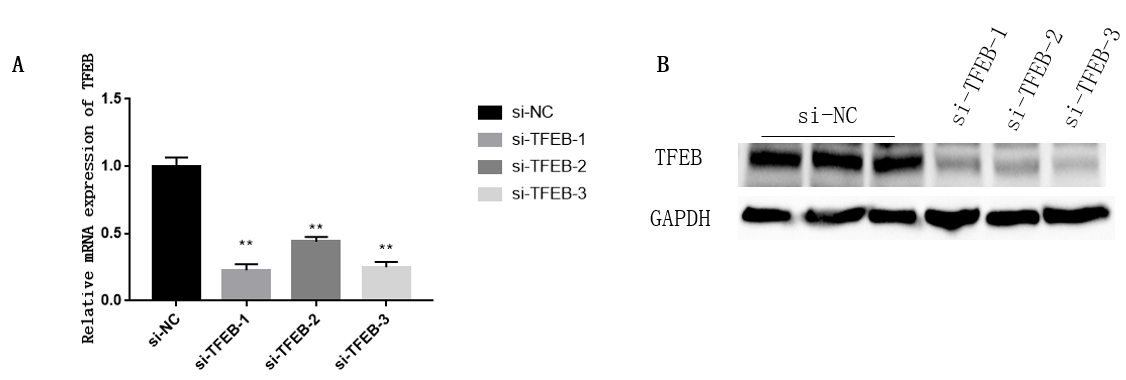


**Supplementary Figure S9.** **TFEB mRNA and protein expressions in HK-2 cells transfected with si-TFEB** (A) Relative *TFEB* mRNA expression in HK-2 cells transfected with si-TFEB. ***P* < 0.01 compared with the si-NC group (*n* = 3/group). (B) Relative TFEB protein levels in HK-2 cells transfected with si-TFEB.
